# Association between cognitive impairment and apolipoprotein A1 or apolipoprotein B levels is regulated by apolipoprotein E variant rs429358 in patients with chronic schizophrenia

**DOI:** 10.18632/aging.203161

**Published:** 2021-06-16

**Authors:** Wenwang Rao, Yunshu Zhang, Keqing Li, Xiang Yang Zhang

**Affiliations:** 1 Institute of Mental Health, Hebei Mental Health Centre, Hebei Province, China; 2 Unit of Psychiatry, Department of Public Health and Medicinal Administration & Institute of Translational Medicine, Faculty of Health Sciences, University of Macau, Macao SAR, China; 3 Department of Sleep Medicine, Hebei Psychiatric Hospital, Hebei Province, China; 4 CAS Key Laboratory of Mental Health, Institute of Psychology, Chinese Academy of Sciences, Beijing, China

**Keywords:** apolipoprotein A1, apolipoprotein B, apolipoprotein E, cognition, schizophrenia

## Abstract

ApoE gene polymorphism may be involved in the change in blood lipid profile and cognitive impairment of the general population. However, few studies explored the effects of ApoE gene polymorphism on blood lipid levels and cognition in schizophrenia. The Repeatable Battery for the Assessment of Neuropsychological Status (RBANS) was employed to evaluate the cognition and the SNPStats was used to investigate the association of *ApoE* rs429358 with schizophrenia. The models of analysis of covariance and multivariate analysis were conducted to investigate the effect of *ApoE* rs429358 on cognition in schizophrenia. Altogether, 637 patients with schizophrenia and 467 healthy controls were recruited in this study. The findings in the case group found that both the ApoA1 and ApoB levels were predictors for RBANS total score (*p* < 0.001 vs. *p* = 0.011), immediate memory (*p* < 0.001 vs. *p* = 0.019), language (*p* < 0.001 vs. *p* = 0.013), attention (*p* < 0.001 vs. *p* < 0.001), except ApoA1 level only was a predictor for visuospatial/constructional (*p* = 0.014) and delayed memory (*p* < 0.001). When the association was examined in different *ApoE* rs429358 genotype subgroups, the association between ApoA1 level and RBANS scores (except for the language score) or between ApoB level and RBANS scores (except for the attention score) was regulated by *ApoE* rs429358. Our results suggest that patients with schizophrenia have broad cognitive impairment compared with healthy controls. For patients with schizophrenia, both ApoA1 and ApoB levels were positively associated with cognition. There was a significant association between ApoA1 or ApoB levels and cognition in schizophrenia, which was regulated by the ApoE rs429358.

## INTRODUCTION

Schizophrenia is a severe long-term mental illness with an estimated lifetime prevalence rate of 0.4–0.88% [1–4], which is associated with serious adverse consequences during the deterioration of the disease, such as high disability [5], premature mortality [6], potential years of life loss [7] and functional and cognitive decline [8]. In addition, previous studies have shown that schizophrenia may lead to a high financial burden [9, 10]. A large number of literatures have revealed that patients with schizophrenia are characterized by changes in blood lipid profiles [11, 12] and various cognitive disorders, including learning, memory, attention, executive function, and information processing [13, 14]. However, the pathophysiological mechanisms of underlying cognitive impairment and blood lipid profile changes in patients with schizophrenia are still unclear.

The human *apolipoprotein* E (*ApoE*) variant originates from two functional polymorphisms (rs429358 and rs7412) in exon 4 of the *ApoE* gene, which can be combined to form three major subtypes (2,3 and 4) [15]. Previous studies have shown that *ApoE* gene polymorphism may affect the level of lipoprotein [16–18] and also affect the development of schizophrenia [19, 20]. For example, one study found a significant association between *ApoE* 3 gene variation and schizophrenia in Asian populations [19]; Another study found a significant association between *ApoE* rs429358 or *ApoE* rs7412 polymorphisms and low-density lipoprotein levels (LDL) in whites and African Americans [21]. In addition, the *ApoE* gene polymorphisms were also associated with cognitive impairment [22]. Some studies have shown that *ApoE* 4 gene mutations were associated with postoperative cognitive impairment [23], as well as certain areas of cognitive function, including episodic memory, global cognitive ability, executive function and perceptual speed [24]. Young people with *ApoE* 4 gene mutations performed better in episodic and working memory, executive function and language fluency [25, 26]. Interestingly, one study found that *ApoE*4 carriers had better language fluency in the 51-65 age group than *ApoE*3 carriers [27].

Apolipoproteins A1(ApoA1) and apolipoproteins B (ApoB) are common apolipoproteins (Apos) related to cholesterol and lipid metabolism [28], and may also be involved in the process of neurodegeneration [29, 30]. ApoA1 is the main protein component of HDL, and a biomarker of cardiovascular disease [31], while ApoB is the main protein including plasma chylomicrons, very-low-density lipoprotein (VLDL) and LDL [32], which can be used as a biomarker of schizophrenia [33]. On the one hand, numerous studies have observed the mixed changes in ApoA1 and ApoB levels in patients with schizophrenia, such as an increase in ApoA1 [31, 34, 35] or ApoB [33, 36, 37] and reduction of ApoA1 [37, 38], or ApoB [35]. On the other hand, ApoA1 and ApoB levels may be associated with cognitive decline [39, 40], which has been indirectly verified by a number of mouse experiments [41, 42]. Specifically, Lewis et al. found that using a triple transgenic mouse model, the over-expression of ApoA1 prevented the development of age-related learning and memory deficits, despite the continued Aβ deposition. Bereczki et al. found that over-expression of human ApoB caused the formation of amyloid plaques and extensive neuronal death in the serum of transgenic mice. In addition, *ApoE* gene polymorphism may affect expression levels of ApoA1 and ApoB [40, 43, 44]. Nevertheless, few studies have been performed on this topic. Some speculations have been proposed. For example, one study has proposed that genetic variation in the coding region of the *ApoE* gene (Apoε2/ε3/ε4) plays a critical role in modulating atherogenic ApoA1/B-containing lipoproteins [45, 46]. Another study has hypothesized that assembly or structure of lipoprotein particles is affected, which in turn may alter the half-lives of their various apolipoprotein components [47].

Numerous empirical results suggested there was an association between *ApoE* gene and Alzheimer's disease [48, 49], between *ApoE* gene and lipid levels [50], as well as between *ApoE* gene and cognition [51]. However, few studies have explored the effects of *ApoE* gene polymorphism on blood lipid levels and cognition in schizophrenia. Therefore, we conducted this study to explore the relationship between cognitive impairment and ApoA1 and ApoB levels in patients with schizophrenia, because it may be altered by the *ApoE* polymorphism rs429358. We hypothesized that the *ApoE* polymorphism rs429358 would lead to the changes in ApoA1 and ApoB levels, thereby playing a role in cognitive impairment in schizophrenia. This study had 3 main purposes: (1) to examine the effect of *ApoE* polymorphism rs429358 on cognitive function of patients with schizophrenia and healthy controls; (2) to investigate the relationship between ApoA1 or ApoB levels and cognitive function in patients with schizophrenia; and (3) to investigate whether the relationship between ApoA1 or ApoB levels and cognitive function is regulated by the *ApoE* polymorphism rs429358.

## METHODS

### Study participants

A total of 637 patients with schizophrenia were recruited from Beijing Hui-Long-Guan hospital, and Hebei Rongjun Hospital in Baoding city near Beijing. All inpatients met the following inclusion criteria:(a) according to the Structure Clinical Interview for DSM-IV (SCID), schizophrenia was diagnosed by two psychiatrists with a Kappa value greater than 0.80; (b) at least 12 months of the course of disease; (c) had a stable dose of oral antipsychotic medicines for at least 12 weeks before entering this study. The average antipsychotic dose (equivalent to chlorpromazine) was 391.72 ± 181.22 mg/day [52–54]. The exclusion criteria were as follows: (a) diagnosis of drug or alcohol abuse/dependence; (b) suffering from major physical diseases, including head injury, epilepsy, cardiovascular disease, cerebrovascular disease, infection, cancer, and diabetes; (c) being pregnant.

A total of 467 healthy controls were recruited from a local community in Haidian District, Beijing. They had no self-reported personal or family history of any mental illness. They were in good physical health, and any subjects with medical illnesses or drug and alcohol abuse/dependence except for tobacco smoking were excluded.

All participants voluntarily participated in this study and gave written informed consent before entering the study. The protocol was approved by the Ethics Committee of Beijing Hui-Long-Guan hospital, and it was implemented in accordance with the Declaration of Helsinki [55].

### Data collection and measures

Well-trained researchers collected general information, socio-demographic characteristics, and medical conditions from pre-designed questionnaires and medical records. The well-validated Chinese version of the Repeatable Battery for the Assessment of Neuropsychological Status (RBANS, Form A) [56–59] was used to assess neurocognitive function by three clinical psychologists. After training, an intraclass correlation coefficient (ICC) was greater than 0.8 among these three psychologists. The RBANS includes 12 subscales, which are used to calculate a total score and 5 age-adjusted index scores (attention, language, delayed memory, immediate memory and visuospatial/construction). The RBANS was evaluated on the same day or the next day of the blood draw. A higher score denotes better ability.

### DNA extraction and SNP genotyping

Genomic DNA was extracted from 5 ml of peripheral venous blood in each sample using standard salting-out procedures [60], and then stored at –80 degree. The *ApoE* polymorphism rs429358 was genotyped by using Matrix-Assisted Laser Desorption/Ionization Time of Flight Mass Spectrometry (MALDI-TOF-MS) (Sequenom Inc., San Diego, CA, USA) according to the protocol [61]. After consulting the NCBI GenBank database for reference sequences, the primers and extension probes were generated. Re-genotyping was performed by trained researchers without knowing the clinical information in 5% randomly selected samples for quality control, with an error rate of less than 0.1%.

### Serum ApoA1 and ApoB levels measurement

Blood sampling and measurement of serum apolipoprotein (ApoA1 and ApoB) levels were described in detail in our previous study [62], which was performed by immunoturbidimetric method (Beijing Leadman Biotechnology, China) on the Olympus AU2700 analyzer [12, 63].

### Statistical analysis

Since all demographic and clinical variables, as well as ApoA1 and ApoB levels were normally distributed in patients and normal controls (Kolmogorov–Smirnov one sample test; all *p* > 0.05), the principal outcome analysis consisted of an independent two-sample *t*-test for continuous variables between cases and controls. Chi-squared (χ^2^) was used for categorical variables between cases and controls. The SNPStats program (a network tool for SNP analysis; https://www.snpstats.net/start.htm) was used to examine the deviation from Hardy-Weinberg disequilibrium (HWD) and genetic model (i.e., dominant, dominant, recessive, and over dominant) analysis for *ApoE* rs429358 [64]. Furthermore, in order to identify the effect of *ApoE* variant rs429358 on susceptibility to schizophrenia, logistic regression analysis was used to control for confounding factors. Since almost no homozygous variant CC genotypes were detected in our study (appropriate 0.8% of patients and 0.5% of healthy controls), we considered the CC and TC genotypes as a group in the following association analysis.

An independent two-sample *t*-test was used to assess between-group differences in continuous variables grouped by genotypes, and Chi-squared (χ2) was used for dichotomous variables. Pearson correlation analysis was used to analyze the correlation between variables, and the partial correlation analysis was carried out with age, gender, education level, BMI and age of onset as covariates. Stepwise regression analysis was used to explore whether there were differences in the relationship between ApoA1 or ApoB levels and RBANS scores among the *ApoE* rs429358 subgroups. In each *ApoE* rs429358 subgroup, the RBANS total or subscale scores were taken as the dependent variables, and ApoA1 and ApoB levels were used as independent variables, adjusting for age, gender, education, BMI and age of onset. Based on the *ApoE* rs429358 genotype subgroups, a two-way analysis of variance (two-way ANCOVA) was used to explore the differences in cognitive scores between patients with schizophrenia and healthy controls. First, the 2 × 2 MANCOVA (genotype × diagnosis) model was used to report the overall p value, and then in this model, the main effects of diagnosis, genotype, and genotype × diagnosis were tested. In this model, diagnosis and *ApoE* rs429358 genotype were used as independent variables, and the score of each cognitive domain and the total score of RBANS were used as dependent variables, with age, gender, education level and BMI as covariates. Bonferroni correction was applied to each test to adjust for multiple tests (P_cor_ = *P* × 6 ), since six comparisons were made between the ApoA1 or ApoB levels and RBANS scores,

Power analysis was conducted using the Quanto software (Version 1.2.3) under log additive, recessive and dominant models, assuming that the prevalence rate of schizophrenia in the population was 1%. All data analyses were performed using SPSS, version 26.0 (IBM SPSS, IBM Corp., Armonk, NY, USA), with a significance level of 0.05 (two-sided).

## RESULTS

### Association analysis of *ApoE* rs429358 with schizophrenia

The demographic and clinical information between schizophrenia and healthy controls are summarized in Table 1. There were significant differences in gender, BMI and age between patients and healthy controls (all *p* < 0.05), which were adjusted as covariates in the following analyses.

**Table 1:** Demographic profiles in patients with schizophrenia and controls (Mean ± SD).

**Variables**	**Association between rs429358 and schizophrenia**				**Association between rs429358 and cognition score**			
	**Cases (*n* = 637)**	**Controls (*n* = 467)**	**t/X^2^**	***P* value**	**Cases (*n* = 415)**	**Controls (*n* = 400)**	**t/X^2^**	***P* value**
Age (year)	47.52 ± 10.61	44.94 ± 13.63	−3.392	0.001	48.19 ± 9.25	45.84 ± 13.37	−2.867	0.004
Sex								
Female (%)	156 (24.5)	274 (58.7)	132.406	<0.001	73 (17.6)	230 (57.5)	138.902	<0.001
Male (%)	481 (75.5)	193 (41.3)			342 (82.4)	170 (42.5)		
Year of education	9.30 ± 6.81	9.66 ± 5.33	0.955	0.340	9.19 ± 6.77	9.40 ± 5.59	0.473	0.636
Body mass index	24.45 ± 4.00	25.14 ± 4.18	2.538	0.011	24.68 ± 3.89	25.41 ± 4.14	2.378	0.018
Age of onset	23.20 ± 5.16	–	–	–	23.12 ± 4.67	–	–	–
Illness of course (year)	24.43 ± 10.52	–	–	–	25.23 ± 9.49	–	–	–
Mean daily dose (mg/day) (chlorpromazine equivalents)	391.72 ± 181.22	–	–	–	388.78 ± 171.19	–	–	–
ApoA1 level (g/L)	1.53 ± 0.38	–	–	–	1.50 ± 0.38	–	–	–
ApoB level (g/L)	0.89 ± 0.24	–	–	–	0.88 ± 0.25	–	–	–
RBANS Total score	–	–	–	–	64.60 ±15.66	80.04 ± 15.12	14.308	<0.001
Immediate memory score	–	–	–	–	58.68 ± 16.82	75.69 ± 17.31	14.219	<0.001
Attention score	–	–	–	–	71.56 ± 18.13	87.44 ± 20.35	11.767	<0.001
Language score	–	–	–	–	81.06 ± 15.45	93.89 ± 13.09	12.810	<0.001
Visuospatial/Constructional score	–	–	–	–	78.00 ± 19.45	79.62 ± 15.59	1.313	0.190
Delayed memory score	–	–	–	–	66.24 ± 19.27	86.25 ± 15.26	16.465	<0.001

The HWD test revealed that the genotype distributions of *ApoE* rs429358 in both schizophrenia and controls were consistent with HWD (case: *p* = 0.52; control: *p* = 1.0; all: *p* = 0.82). We did not observe a significant difference in *ApoE* rs429358 genotype distributions under the inheritance model (all *p* > 0.05). After adjusting for age, gender and BMI, there were still no significant differences in the distribution of alleles and genotypes (all *p* > 0.05).

### Cognition between patients and controls

A total of 815 subjects (415 patients and 400 healthy controls) completed the cognitive assessment. Except for the years of education, there were significant differences in gender, age and BMI between patients and controls (all *p* < 0.05). Therefore, these significant variables were used as covariates in the following analyses. The ANOVA analysis indicated that RBANS total score and its subscale scores (except for visuospatial/constructional score) of healthy controls were significantly greater than those of patients with schizophrenia (all *p* < 0.05). Moreover, after adjusting for covariates including sex, age and BMI, the ANCOVA analysis showed that there were significant differences in immediate memory score (*F* = 136.97, *p* < 0.001), language score (*F* = 121.80, *p* < 0.001), attention score (*F* = 117.13, *p* < 0.001), delayed memory score (*F* = 197.49, *p* < 0.001) and RBANS total score (*F* = 159.94, *p* < 0.001) between the two groups.

### Effects of *ApoE* rs429358 on cognition in patients and controls

Two-way ANOVA analysis showed that diagnosis had a significant effect on all RBANS scores except for visuospatial/constructional score. However, genotype and genotype X diagnosis had no significant effect on any RBANS scores (all *p* > 0.05; Table 2). When controlling for sex, age BMI and education, the above results did not change significantly. In addition, in the patient group, there was no significant difference in RBANS scores between the *ApoE* rs429358 genotype groups (all *p* > 0.05).

**Table 2:** Comparisons among the RBANS total and five subscale scores by diagnostic and genotypic groupings (Mean ± SD).

**RBANS scores**	**Cases**		**Controls**		**Diagnosis**	**Genotype**	Diagnosis ^*^ genotype
	**TT (*n* = 315)**	**CC + CT (*n* = 53)**	**TT (*n* = 336)**	**CC + CT (*n* = 64)**	**F (*p* value)**	**F (*p* value)**	**F (*p* value)**
Immediate memory	56.67 ± 15.50	61.17 ± 20.10	75.91 ± 17.33	74.52 ± 17.28	86.860 (<0.001)	0.387 (0.534)	2.084 (0.149)
Attention	70.13 ± 17.94	72.09 ± 15.85	87.76 ± 20.38	85.75 ± 20.31	65.908 (<0.001)	<0.001 (0.990)	1.064 (0.303)
Language	80.68 ± 15.23	81.42 ± 16.20	93.78 ± 12.60	94.47 ± 15.49	83.125 (<0.001)	0.248 (0.619)	<0.001 (0.986)
Visuospatial/Constructional	76.68 ± 18.60	76.68 ± 20.12	79.61 ± 15.51	79.67 ± 16.15	0.709 (0.400)	0.774 (0.379)	0.716 (0.398)
Delayed memory	64.88 ± 18.70	68.28 ± 20.59	86.39 ± 15.08	85.52 ± 16.29	125.553 (<0.001)	0.536 (0.464)	1.529 (0.217)
Total	63.48 ± 14.24	65.00 ± 18.97	80.16 ± 14.97	79.44 ± 16.04	104.854 (<0.001)	0.069 (0.792)	0.544 (0.461)

### Genotype effects on serum ApoA1 and ApoB levels between patients

ApoA1 levels were available for 507 patients, while ApoB levels were available for 505 patients. There were no ApoA1 and ApoB levels available for healthy controls. There was a significant difference in serum ApoA1 levels between the *ApoE* rs429358 genotype groups, showing that patients with TT genotype had higher ApoA1 levels than patients with CT+CC genotype (*p* = 0.010, Table 3). After adjusting the BMI, the difference remained significant (*p* = 0.005). In addition, there was no significant difference in ApoB levels between *ApoE* rs429358 genotype groups (*p* = 0.545).

**Table 3:** Demographic and clinical information according to *ApoE* rs429358 genotype of cases and controls in a Chinese sample.

**Variables**	**Cases (*n* = 506)**				**Health Controls (*n* = 421)**			
	**CT+CC (*n* = 73)**	**TT (*n* = 433)**	**t/X^2^**	***P* value**	**CT+CC (*n* = 67)**	**TT (*n* = 354)**	**t/X^2^**	***P* value**
Age (year)	47.45 ± 10.04	47.36 ± 9.66	−0.077	0.939	46.15 ± 13.14	46.16 ± 13.27	0.006	0.995
Sex								
Female (%)	19 (26.0)	91 (21.0)	0.922	0.337	38 (56.7)	210 (59.3)	0.158	0.691
Male (%)	54 (74.0)	342 (79.0)			29 (43.3)	144 (40.7)		
Year of education	10.21 ± 10.91	8.72 ± 2.60	−1.159	0.250	10.80 ± 11.52	9.14 ± 3.32	−1.164	0.248
Body mass index	23.55 ± 4.12	24.73 ± 3.85	2.131	0.034	25.00 ± 3.58	25.41 ± 4.20	0.734	0.464
Age of onset	23.34 ± 5.58	22.98 ± 4.77	−0.587	0.558	–	–	–	–
Illness of course (year)	24.11 ± 9.56	24.53 ± 10.07	0.335	0.738	–	–	–	–
Mean daily dose (mg/day) (chlorpromazine equivalents)	413.29 ± 178.79	380.97 ± 159.69	−1.166	0.245	–	–	–	–
ApoA1 level (g/L)	1.38 ± 0.40	1.52 ± 0.37	2.586	0.010	–	–	–	–
ApoB level (g/L)	0.86 ± 0.26	0.88 ± 0.26	0.606	0.545	–	–	–	–

### Relationship between serum ApoA1 levels and cognition in patients

Pearson correlation analysis showed a significant association between ApoA1 level and RBANS total score (*r* = 0.343, *n* = 318, *p* < 0.001, *p*_cor_ < 0.006), immediate memory (*r* = 0.251, *n* = 318, *p* < 0.001, *p*_cor_ < 0.006), language (*r* = 0.243, *n* = 318, *p* < 0.001, *p*_cor_ < 0.006), attention (*r* = 0.401, *n* = 318, *p* < 0.001, *p*_cor_ < 0.006), visuospatial/constructional (*r* = 0.230, *n* = 318, *p* < 0.001, *p*_cor_ < 0.006) or delayed memory (*r* = 0.261, *n* = 318, *p* < 0.001, *p*_cor_ < 0.006). Furthermore, after controlling for age, gender, BMI, years of education, and age of onset, ApoA1 level was still correlated with the RBANS total score (*r* = 0.287, *n* = 211, *p* < 0.001, *p*_cor_ < 0.006), immediate memory (*r* = 0.252, *n* = 211, *p* < 0.001, *p*_cor_ < 0.006), language (*r* = 0.321 *n* = 211, *p* < 0.001, *p*_cor_ < 0.006), attention (*r* = 0.321, *n* = 211, *p* < 0.001, *p*_cor_ < 0.006), visuospatial/constructional (*r* = 0.168, *n* = 211, *p* = 0.014, *p*_cor_ = 0.084) and delayed memory domains (*r* = 0.198, *n* = 211, *p* = 0.004, *p*_cor_ = 0.024). In addition, linear regression analyses identified that ApoA1 level was a predictor for RBANS total score (*t* = 4.359, *p* < 0.001, *p*_cor_ < 0.006), immediate memory (*t* = 3.790, *p* < 0.001, *p*_cor_ < 0.006), language (*t* = 4.924, *p* < 0.001, *p*_cor_ < 0.006), attention (*t* = 4.925, *p* < 0.001, *p*_cor_ < 0.006), visuospatial/constructional (*t* = 2.474, *p* = 0.014, *p*_cor_ = 0.084) and delayed memory domains (*t* = 2.934, *p* < 0.001, *p*_cor_ < 0.006).

### Relationship between serum ApoB levels and cognition in patients

The ApoB level was associated with RBANS total score (*r* = 0.245, *n* = 316, *p* < 0.001, *p*_cor_ < 0.006), immediate memory (*r* = 0.211, *n* = 316, *p* < 0.001, *p*_cor_ < 0.006), language (*r* = 0.185, *n* = 316, *p* = 0.001, *p*_cor_ < 0.006), attention (*r* = 0.315, *n* = 316, *p* < 0.001, *p*_cor_ < 0.006), visuospatial/constructional (*r* = 0.166, *n* = 316, *p* = 0.003, *p*_cor_ = 0.018) and delayed memory (*r* = 0.181, *n* = 316, *p* = 0.001, *p*_cor_ = 0.006). After controlling for age, gender, BMI, years of education and age of onset, the ApoB level was still correlated with RBANS total score (*r* = 0.175, *n* = 209, *p* = 0.011, *p*_cor_ = 0.066), immediate memory (*r* = 0.161, *n* = 209, *p* = 0.019, *p*_cor_ = 0.114), language (*r* = 0.171, *n* = 209, *p* = 0.013, *p*_cor_ = 0.078), and attention (*r* = 0.334, *n* = 209, *p* < 0.001, *p*_cor_ < 0.006). In addition, linear regression analyses identified that the ApoB level was a predictor for RBANS total score (*t* = 2.574, *p* = 0.011, *p*_cor_ = 0.066), immediate memory (*t* = 2.357, *p* = 0.019, *p*_cor_ = 0.114), language (*t* = 2.517, *p* = 0.013, *p*_cor_ = 0.078), and attention (*t* = 5.120, *p* < 0.001, *p*_cor_ < 0.006).

### Genotype effects on serum ApoA1 level and cognition in patients

In the T homozygote group, Pearson correlation analysis showed a significant positive association between ApoA1 level and RBANS total score, (*r* = 0.384, *n* = 234, *p* < 0.001, *p*_cor_ < 0.006), immediate memory index (*r* = 0.233, *n* = 234, *p* < 0.001, *p*_cor_ < 0.006), visuospatial/constructional index (*r* = 0.260, *n* = 234, *p* < 0.001, *p*_cor_ < 0.006), language index (*r* = 0.268, *n* = 234, *p* < 0.001, *p*_cor_ < 0.006), attention index (*r* = 0.408, *n* = 234, *p* < 0.001, *p*_cor_ < 0.006) and delayed memory index (*r* = 0.253, *n* = 234, *p* < 0.001, *p*_cor_ < 0.006). Moreover, after controlling for age, gender, BMI, years of education and age of onset, the ApoA1 level still positively associated with the RBANS total score (*r* = 0.398, *n* = 163, *p* < 0.001, *p*_cor_ < 0.006), immediate memory index (*r* = 0.308, *n* = 163, *p* < 0.001, *p*_cor_ < 0.006), visuospatial/constructional index (*r* = 0.198, *n* = 163, *p* = 0.011, *p*_cor_ = 0.066), language index (*r* = 0.357, *n* = 163, *p* < 0.001, *p*_cor_ < 0.006), attention index (*r* = 0.338, *n* = 163, *p* < 0.001, *p*_cor_ < 0.006) and delayed memory index (*r* = 0.245, *n* = 163, *p* = 0.001, *p*_cor_ = 0.006). Further regression analysis confirmed that ApoA1 level was significantly associated with RBANS total score (*t* = 6.272, *p* < 0.001, *p*_cor_ < 0.006), immediate memory index (*t* = 4.789, *p* < 0.001, *p*_cor_ < 0.006), visuospatial/constructional index (*t* = 2.704, *p* = 0.008, *p*_cor_ = 0.048), language index (*t* = 5.215, *p* < 0.001, *p*_cor_ < 0.006), attention index (*t* = 5.444, *p* < 0.001, *p*_cor_ < 0.006) and delayed memory index (*t* = 3.672, *p* < 0.001, *p*_cor_ < 0.006).

In the C allele carriers, ApoA1 level was significantly associated with RBANS total score (*r* = 0.448, *n* = 42, *p* = 0.003, *p*_cor_ = 0.012), immediate memory index (*r* = 0.522, *n* = 42, *p* < 0.001, *p*_cor_ < 0.006), visuospatial/constructional index (*r* = 0.357, *n* = 42, *p* = 0.020, *p*_cor_ = 0.120), language index (*r* = 0.542, *n* = 42, *p* < 0.001, *p*_cor_ < 0.006), attention index (*r* = 0.608, *n* = 42, *p* < 0.001, *p*_cor_ < 0.006) and delayed memory index (*r* = 0.440, *n* = 42, *p* = 0.004, *p*_cor_ = 0.024). After controlling for age, gender, BMI, duration of education and age of onset, ApoA1 level was only associated with language index (*r* = 0.431, *n* = 25, *p* = 0.025, *p*_cor_ = 0.150).

### Genotype effects on serum ApoB level and cognition in patients

In the T homozygote group, there was a significant association between ApoB levels and the RBANS total score (*r* = 0.260, *n* = 233, *p* < 0.001, *p*_cor_ < 0.006), immediate memory index (*r* = 0.222, *n* = 233, *p* = 0.001, *p*_cor_ = 0.006), visuospatial/constructional index (*r* = 0.152, *n* = 233, *p* = 0.020, *p*_cor_ = 0.120), language index (*r* = 0.161, *n* = 233, *p* = 0.014, *p*_cor_ = 0.084), attention index (*r* = 0.293, *n* = 233, *p* < 0.001, *p*_cor_ < 0.006) and delayed memory index (*r* = 0.173, *n* = 233, *p* = 0.008, *p*_cor_ = 0.048). When controlling for age, gender, BMI, duration of education and age of onset, RBANS total score (*r* = 0.231, *n* = 162, *p* = 0.003, *p*_cor_ = 0.018), immediate memory index (*r* = 0.172, *n* = 162, *p* = 0.028, *p*_cor_ = 0.168), language index (*r* = 0.170, *n* = 162, *p* = 0.029, *p*_cor_ = 0.174), and attention index (*r* = 0.332, *n* = 162, *p* < 0.001, *p*_cor_ < 0.006) remained significant. Furthermore, regression analysis showed that ApoB level was significantly associated with the RBANS total score (*t* = 2.971, *p* = 0.003, *p*_cor_ = 0.018), immediate memory index (*t* = 2.333, *p* = 0.021, *p*_cor_ = 0.126), language index (*t* = 2.540, *p* = 0.012, *p*_cor_ = 0.072) and attention index (*t* = 5.012, *p* < 0.001, *p*_cor_ < 0.006).

In the C allele carriers, Pearson correlation analysis showed a significant association between ApoB level and immediate memory index (*r* = 0.395, *n* = 41, *p* = 0.010, *p*_cor_ = 0.060) or attention index (*r* = 0.582, *n* = 41, *p* < 0.001, *p*_cor_ < 0.006). After controlling for age, gender, BMI, years of education and age of onset, only the attention index was still significantly associated with ApoB level (*r* = 0.481, *n* = 24, *p* = 0.013, *p*_cor_ = 0.078).

## DISCUSSION

To our best knowledge, this is the first report to explore the relationship between *ApoE* rs429358 and cognitive impairment in patients with schizophrenia. This study had several main findings. (1) *ApoE* rs429358 may not be associated with susceptibility to schizophrenia. (2) In patients, serum ApoA1 level was significantly higher in the *ApoE* rs429358 TT genotype group than that in the CT + CC genotype group. (3) Except for the visuospatial/constructional domain, RBANS total score and all other domains of patients with schizophrenia were significantly lower than those of healthy controls. (4) *ApoE* rs429358 genotype was not associated with any cognitive performance shown on the RBANS. (5) Except for the visuospatial/constructional domain delayed memory domain, the RBANS total score and the other 3 domains were correlated with serum ApoA1 and ApoB levels. (6) The association between serum ApoA1 level and RBANS scores (except language score) or between serum ApoB level and RBANS scores (except attention score) was regulated by *ApoE* rs429358.

This study did not find any association between *ApoE* rs429358 and schizophrenia, which is consistent with previous studies [65–67]. Nevertheless, an early meta-analysis showed an association of *ApoE* ε3 with schizophrenia in Asian populations [19], but not in other populations. Meanwhile, a highly significant association was found between *ApoE* genotype and schizophrenia in the Chinese population [68]. Moreover, an association study and meta-analysis revealed an association between schizophrenia and *ApoE* ε2ε3 genotype in French male samples but not in the entire French sample [69]. In addition, there was an association between undifferentiated type of schizophrenia and *ApoE* ε3/ε3 genotype in the Serbian population [70]. Interestingly, both *ApoE* ε3 and *ApoE*-219G haplotypes increased the risk of schizophrenia in siblings [71]. By comparing the above studies, we speculate that these different results between our current study and other studies may be partly due to differences in population, sample composition, types of schizophrenia and synergy/interaction with other variants.

The Apolipoprotein E (*ApoE*) gene (4 exons and 3 introns) plays a key role in receptor-mediated endocytosis of lipoproteins in the brain [72] and affects downstream proteins, such as brain-derived neurotrophic factor (BDNF) [73, 74], which may be involved in neuropsychiatric genetics [74, 75], especially in cognition-related diseases [76]. In addition, ApoB level are associated with the low-density lipoprotein receptor (LDLR) [77, 78], which is expressed in brain capillary endothelial cells and astrocytes, and plays an important role in cholesterol and Aβ clearance [79]. ApoA1 level plays a major role in cholesterol transport in the central nervous system (CNS) [80, 81]. Brain cholesterol is considered to be involved in the development of blood-brain barrier [82–84]. Blood-brain barrier damage is associated with the occurrence and development of cognitive impairment [85].

It is well-known that *ApoE* gene polymorphism was associated with cognitive decline, which has been confirmed by some studies [86, 87], mouse experiments [88, 89], clinical studies [90, 91] and meta-analysis [24]. Moreover, previous studies have revealed an association between the *ApoE* variant rs429358 (rather than rs7412) and cognitive decline in the aging population [51, 92, 93]. However, our research did not support this association. One possible explanation is that abnormalities caused by schizophrenia may affect the impact of rs429358 on cognitive function. In addition, a study found that schizophrenia patients with *ApoE*ε4 carriers had significantly lower verbal memory score assessed by the Brief Assessment of Cognition in Schizophrenia (BACS) [22]. Another study showed an interaction between the *ApoE* ε4 allele, first-episode psychosis (FEP), and the improvement in verbal memory over time measured by California Verbal Learning Test (CVLT) [94]. These inconsistent findings of cognitive deficits in patients with schizophrenia may be partly due to the combined effects of different cognitive measurement tools and *ApoE* genotypes.

Interestingly, to our best knowledge, this is a first report showing that ApoA1 level was positively associated with all domains of RBANS scores in patients with schizophrenia, while ApoB level was correlated with the RBANS total score and other 3 domains, except for visuospatial/constructional domain and delayed memory domain. Similarly, a study reported that ApoA1 level was independently associated with cognitive impairment as shown by the Mini Mental Status Examination (MMSE) in elderly men [95]. Another study of the Swedish Adoption Twin Study of Aging of 50 years and older demonstrated that ApoA1 level was significantly associated with perceptual speed in women, while ApoB level was associated with perceptual speed in men and verbal ability in women [39]. These consistent results may suggest that the impacts of ApoA1 and ApoB levels on cognitive function may not be affected by schizophrenia. Moreover, the association between serum ApoA1 level and RBANS scores (except for the language score) or between serum ApoB level and RBANS scores (except for the attention score) was regulated by the *ApoE* rs429358 genotype. We speculate that this regulatory effect of *ApoE* rs429358 may be achieved by controlling the expression levels of ApoA1 and ApoB. Although there was no significant difference in ApoB levels between different *ApoE* rs429358 genotype groups in our study, many studies have found that *ApoE* gene polymorphisms affect the expression levels of ApoA1 and ApoB [40, 43, 44].

This study has several limitations. First, the controls were different from the cases in several key demographics (like age, sex and BMI), which may make the case vs control analysis less relevant. Although we adjusted these different key demographics in the statistical analysis models, it may still lead to bias in the statistical analysis due to these different variables between cases and controls, which should be remedied in future studies that will select the controls to match the cases. Second, owing to the cross-sectional study design, the causality between *ApoE* rs429358, ApoA1 and ApoB levels, and cognition impairment in patients with schizophrenia was not directly revealed. Third, compared with typical psychiatric outpatients or first-episode patients with schizophrenia and aboriginal patients, the inpatients in this study had more severe psychopathology, cognitive decline and longer duration of disease and antipsychotic treatment, which may limit the generalization of our findings of this study. Fourth, *ApoE* rs429358 has a rare CC genotype. Therefore, this study combined CC and TC genotypes as a group for association analysis, which may cause uncertainty in the results. Fifth, in this study, we only examined the effects of a single genetic polymorphism, and it is necessary to detect other functional variants of the *ApoE* gene (e.g., rs7412), because other polymorphisms, haplotypes, gene interaction or gene-environment interaction may be associated with schizophrenia or with cognitive dysfunction in patients with schizophrenia. Sixth, the RBANS is unable to assess all cognitive domains that may be altered in patients, such as motor ability or executive function. Although the RBANS scale has been well verified for the Chinese general population and patients with schizophrenia, its lacks of norm value restricts its use in the Chinese population.

In summary, the *ApoE* rs429358 gene polymorphism did not directly affect the susceptibility to schizophrenia and cognitive function of schizophrenia. However, the serum ApoA1 and ApoB levels were positively correlated with the degree of cognitive function in patients with schizophrenia, indicating that serum ApoA1 and ApoB levels may be biomarkers of general cognitive function in schizophrenia patients. The association between serum ApoA1 or ApoB levels and cognitive impairment in patients with schizophrenia were regulated by the existence of *ApoE* rs429358 polymorphism. However, due to the limited sample size and relatively low statistical power, our findings are still preliminary. Therefore, future studies are needed to confirm our current findings in larger samples from different ethnics, and the biological mechanisms of cognitive impairments in schizophrenia involved in *ApoE* rs429358 should also be further studied.
